# R-spondin 2 mediates neutrophil egress into the alveolar space through increased lung permeability

**DOI:** 10.1186/s13104-020-4930-8

**Published:** 2020-02-04

**Authors:** S. R. Jackson, M. F. D. M. Costa, C. F. Pastore, G. Zhao, A. I. Weiner, S. Adams, G. Palashikar, K. Quansah, K. Hankenson, D. R. Herbert, A. E. Vaughan

**Affiliations:** 1 Department of Biomedical Sciences, School of Veterinary Medicine, University of Pennsylvania, 3800 Spruce St., Old Vet 372E, Philadelphia, PA 19104 USA; 2 Institute for Regenerative Medicine, University of Pennsylvania, Philadelphia, PA 19104 USA; 3 Department of Orthopaedic Surgery, University of Michigan School of Medicine, Ann Arbor, MI 48109 USA

**Keywords:** R-spondin 2, Neutrophil migration, Lung permeability, Lung endothelial barrier, BALF, Wnt signaling, FITC-dextran, Alveolar space

## Abstract

**Objective:**

R-spondin 2 (RSPO2) is required for lung morphogenesis, activates Wnt signaling, and is upregulated in idiopathic lung fibrosis. Our objective was to investigate whether RSPO2 is similarly important in homeostasis of the adult lung. While investigating the characteristics of bronchoalveolar lavage in RSPO2-deficient (RSPO2^−/−^) mice, we observed unexpected changes in neutrophil homeostasis and vascular permeability when compared to control (RSPO2^+/+^) mice at baseline. Here we quantify these observations to explore how tonic RSPO2 expression impacts lung homeostasis.

**Results:**

Quantitative PCR (qPCR) analysis demonstrated significantly elevated myeloperoxidase (MPO) expression in bronchoalveolar lavage fluid (BALF) cells from RSPO2^−/−^ mice. Likewise, immunocytochemical (ICC) analysis demonstrated significantly more MPO+ cells in BALF from RSPO2^−/−^ mice compared to controls, confirming the increase of infiltrated neutrophils. We then assessed lung permeability/barrier disruption via Fluorescein Isothiocyanate (FITC)-dextran instillation and found a significantly higher dextran concentration in the plasma of RSPO2^−/−^ mice compared to identically treated RSPO2^+/+^ mice. These data demonstrate that RSPO2 may be crucial for blood-gas barrier integrity and can limit neutrophil migration from circulation into alveolar spaces associated with increased lung permeability and/or barrier disruption. This study indicates that additional research is needed to evaluate RSPO2 in scenarios characterized by pulmonary edema or neutrophilia.

## Introduction

R-spondins (RSPO1–4) are a family of four secreted potentiators of the canonical Wnt/β-catenin signaling pathway, and they act as ligands for leucine-rich repeat-containing G-protein coupled receptors (LGRs) 4–6 [1]. RSPO2 is required for normal lung morphogenesis [2]. Disruption of the RSPO2 locus results in severely hypoplastic lungs at birth that exhibit more than a 50% reduction in weight [2]. Notably, the overall structures and relative ratios of differentiated pulmonary epithelial cells remain unchanged, indicating that hypoplasia is not reflective of failed differentiation, but rather reduced proliferation [2]. RSPO2 expression is largely restricted to the lung mesenchyme, suggesting a paracrine effect on the developing epithelium [2]. The physiological relevance of RSPO2 expression in adult lungs is unclear, although RSPO2 has been demonstrated to play a role in idiopathic lung fibrosis and can be used as a biomarker for lung cancer [3, 4]. Whether RSPO2 plays any role in an inflammatory context (i.e., in leukocyte homeostasis or plasma extravasation) has not been investigated.

Neutrophils are bone marrow-derived polymorphonuclear leukocytes, present in the systemic circulation, that respond to inflammation, including sterile injuries (e.g. hypoxia/reperfusion) and invading pathogens [5–7]. While neutrophils play a significant role as immediate responders, their recruitment and activation are highly regulated to protect tissues from unintended effects [8]. This is particularly true in the lung microenvironment, where damage to the endothelium or epithelium from pathogenic or sterile injury can result in neutrophil migration into the alveolar space [8]. Excessive damage to alveolar structures can result in edema, gas-exchange compromise and death [9, 10]. The loosening of endothelial cell junctions and the migration of inflammatory cells—including neutrophils—across the damaged endothelial barrier plays a key role in the pathophysiology of diseases such as acute respiratory distress syndrome (ARDS), cancer, and other inflammatory pathologies [11, 12]. Although the canonical mechanisms governing diapedesis and leukocyte trafficking have been well described, mechanistic variations dependent on the organ milieu and inflammatory status can occur [12].

While our initial goal was to investigate a role for RSPO2 in lung repair, we observed that RSPO2 deletion in the adult lung results in aberrant neutrophil accumulation in the luminal space without a deliberate injury. Recognizing that neutrophils are normally restricted to blood vessels and that endothelial stress/damage can activate and recruit neutrophils into the interstitium or the alveolar parenchyma [13], we found that RSPO2^−/−^ mice had significant lung barrier dysfunction. This work uncovers an important and previously unrecognized role for RSPO2 in the adult lung in regulation of neutrophil homeostasis and lung endothelial barrier maintenance.

## Main text

### Methods

#### Animals and treatment

For all experiments, mice between 6 to 8 weeks of age and 17 g to 20 g were used including males and females in equal proportions. The following strains were utilized: Inducible Cre (UBC-CreERT2) [14] (The Jackson Laboratory Stock #007001), RSPO2 flox (a gift from Dr. Kurt Hankenson, University of Michigan), and C57BL/6 mice. No statistical method was used to predetermine sample size in any of the animal studies. The experiments were not randomized, and the investigators were not blinded to allocation during the experiments and outcome assessments.

#### Cre recombination in vivo

Mice were injected intraperitoneally with tamoxifen (TM) in 100 µl of Mazola^®^ corn oil (1 mg/g body wt) once per day, every other day for a total of 3 doses. For all analyses, tissues were collected 2 days after the last TM injection.

#### Animal euthanasia

Mice were placed into a closed chamber and exposed to isoflurane (Midwest Veterinary Supply) applied to compacted cotton balls until roughly 1 min after breathing stopped, followed by cervical dislocation, as approved by IACUC.

#### Fibroblast isolation

Fibroblasts were isolated according to our general lung cell isolation protocol previously described [15] with slight modifications: 1 × 10^7^ disaggregated lung cells were plated into a Corning^®^ 6-well clear polystyrene flat-bottom microplate (Millipore Sigma) in DMEM + 20% CC + P/S and grown in a 37 °C incubator for 9 days without passaging with media changes on days 3 and 6 before harvesting cells for mRNA or ICC analysis. For Cre-induced recombination in cultured fibroblasts, cells were treated with (4 µM) of 4-Hydroxytamoxifen (4-OHT) dissolved in dimethyl sulfoxide (DMSO; Santa Cruz Biotechnology), once per day, every other day for 5 days. For qPCR and ICC analyses, fibroblast samples were collected 2 days after the last 4-OHT treatment.

#### BALF collection

BALF was collected according to the protocol previously described [16], followed by cytospin preparation.

#### Cytospins

For both cultured fibroblasts and BALF, cells were centrifuged at 570×*g* for 5 min, followed by aspiration of supernatant, and cell pellets were resuspended in 1 ml of PBS solution and fixed onto slides (Fisherbrand™) at 570 rpm for 4 min on a Cytospin 2 (Shandon).

#### Antibody staining

Immunostaining was performed as previously described [16]. The following primary antibodies were used: goat anti-myeloperoxidase (MPO) (1:200 dilution; R&D Systems), rabbit anti-RSPO2 (1:200 dilution; Proteintech). The following secondary antibodies were used: Alexa Fluor 488-conjugated donkey anti-goat (1:1000, Thermo Fisher Scientific), Alexa Fluor 568-conjugated donkey anti-rabbit (1:1000, Thermo Fisher Scientific).

#### Quantification of immunostaining

Mosaic images of cytospins were generated from multiple 20 X fields on an upright fluorescence microscope (Leica DMi8) and tiled in LAS X software. The number of cells staining positive for the relevant antibody was manually counted and calculated as a fraction of total DAPI + cells. We quantified at least three fields per slide, each containing ≥ 300 individual cells.

#### Quantitative PCR (qPCR) analysis

RNA was isolated using the RNeasy™ (Qiagen) kit. mRNA was revered transcribed into cDNA using iScript™ Reverse Transcription Supermix (BioRad). Total RNA input for cDNA synthesis was standardized within each experiment to the RNA isolate with the lowest concentration as measured by Nanodrop (Thermo Fisher Scientific). RT-PCR reactions were performed using SsoAdvanced™ Universal SYBR^®^ Green Supermix (Biorad) and run on an Applied Biosystems QuantStudio 6 Real-Time PCR System (Thermo Fisher Scientific).

#### FITC-dextran permeability assay

The permeability assay was performed as described in the literature [17, 18]. Mice were anaesthetized with isoflurane and administered 40 µl FITC-dextran (10 mg/kg body weight) intranasally. After a 30-min wait to allow FITC-dextran to circulate in the blood, blood was collected via cardiac puncture, and fluorescence intensity was determined using a spectrophotometer (BioTek).

#### Statistical analysis

All statistical calculations were performed using GraphPad Prism. Mann–Whitney test was used to determine significance. A P value of less than 0.05 was considered significant.

#### PCR and qPCR primers

##### Genotyping primers

Rspo2-floxA-Forward: ACTCTTACTGCCTGGGATCCTCATT

Rspo2-floxB-Reverse: CTTCTTCTGAGCACCATCTGC

##### qPCR primers

GAPDH Forward: AGGTCGGTGTGAACGGATTTG

GAPDH Reverse: TGTAGACCATGTAGTTGAGGTCA

RPL37 Forward: CTCGGAGGTTACGGGACTC

RPL37 Reverse: CTTGCCCTCGTAGGTAATGGG

RPL19 Forward: ATG TAT CAC AGC CTG TAC CTG

RPL19 Reverse: TTC TTG GTC TCT TCC TCC TTG

MPO Forward: AGTTGTGCTGAGCTGTATGGA

MPO Reverse: CGGCTGCTTGAAGTAAAACAGG

RSPO2 Forward: AGACGCAATAAGCGAGGTGG

RSPO2 Reverse: CTGCATCGTGCACATCTGTT

### Results

#### Infiltration of neutrophils into bronchoalveolar lavage fluid following RSPO2 deletion

**Fig. 1:**
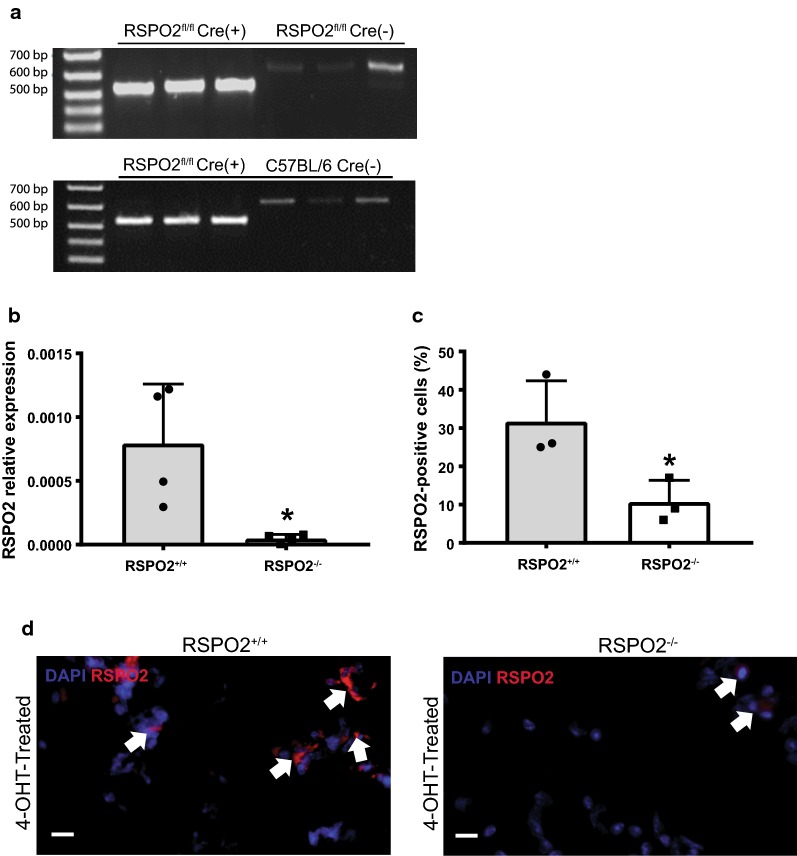
Validation of RSPO2 deletion. **a** RSPO2 gene expression in lung homogenates from RSPO2fl/fl;UBC-Cre-ERT2(+), RSPO2fl/fl;UBC-Cre-ERT2(−), and C57BL/6 mice 48 h post-TM treatment. Cre-recombination of the loxP sites yields a 512 bp fragment, whereas the wild type allele yields a non-specific 600 bp fragment. **b** qPCR analysis of RSPO2 expression in the cultured fibroblasts isolated from the lungs of RSPO2^−/−^ and RSPO2^+/+^ mice. **c**, **d** Quantification of immunocytochemical evidence of RSPO2 expression in fibroblasts isolated from RSPO2^−/−^ and RSPO2^+/+^ mice. An arrow indicates examples of DAPI/RSPO2-double stained cells. Representative images are shown from RSPO2^+/+^ mice (n = 3) and RSPO2^−/−^ mice (n = 3) samples. * = A P value of less than 0.05 was considered significant

**Fig. 2:**
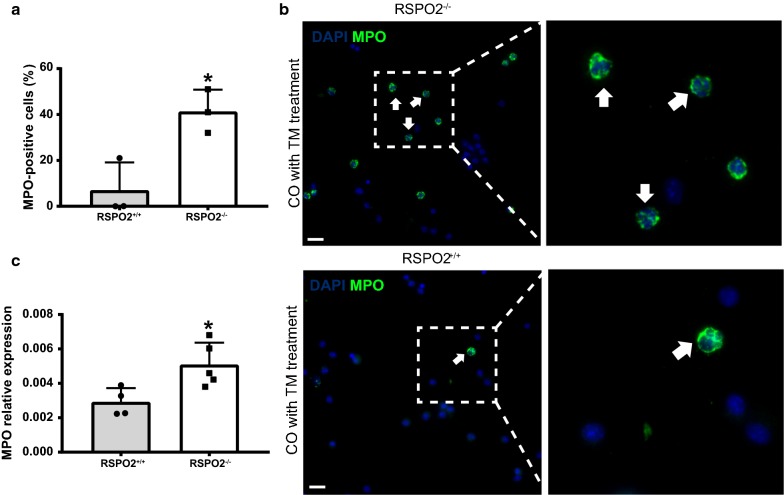
RSPO2 deficiency promotes neutrophil accumulation. **a**, **b** Quantification of ICC of MPO + cells in the BALF of RSPO2^+/+^ mice and RSPO2^−/−^ mice 48 h after TM administration. An arrow indicates examples of DAPI/MPO-double stained cells. **c** qPCR analysis demonstrating a similar increase in MPO transcript in cells present in BALF of RSPO2^−/−^ mice. Representative images are shown from RSPO2^+/+^ mice (n = 3) and RSPO2^−/−^ mice (n = 3) samples. * = A P value of less than 0.05 was considered significant

Given that tissue repair often recapitulates features of embryonic development, where RSPO2 is critical, we generated UBC-CreERT2/RSPO2^flox/flox^ mice to pursue the hypothesis that RSPO2 deletion would impact lung regeneration. We first confirmed successful recombination of the RSPO2 allele in adult mice after TM treatment (Fig. 1a). Additionally, we isolated lung fibroblasts from these animals, treated with 4-OHT in vitro to induce recombination, and confirmed reduction in RSPO2 transcript via qPCR and immunostaining (Fig. 1b–d). Before the originally planned injury experiments were initiated, we examined the lavage fluid of RSPO2 deleted mice to ensure normal levels of immune cells as determined by cytospin cell analysis. Unexpectedly, we observed MPO-expressing cells, a definitive neutrophil marker [19], in the BALF of RSPO2^−/−^ mice at a significantly higher percentage compared to RSPO2^+/+^ mice (Fig. 2a, b). qPCR analysis demonstrated significantly higher MPO expression in BALF cells in RSPO2^−/−^ mice compared to RSPO2^+/+^ mice (Fig. 2c), confirming the increase of infiltrated neutrophils. This indicates RSPO2^−/−^ mice exhibit elevated neutrophil egress into the alveolar space compared to RSPO2^+/+^ mice in terms of both increased MPO-expressing cells and higher MPO mRNA expression in BALF cells.

#### RSPO2 deletion increases lung barrier permeability

**Fig. 3:**
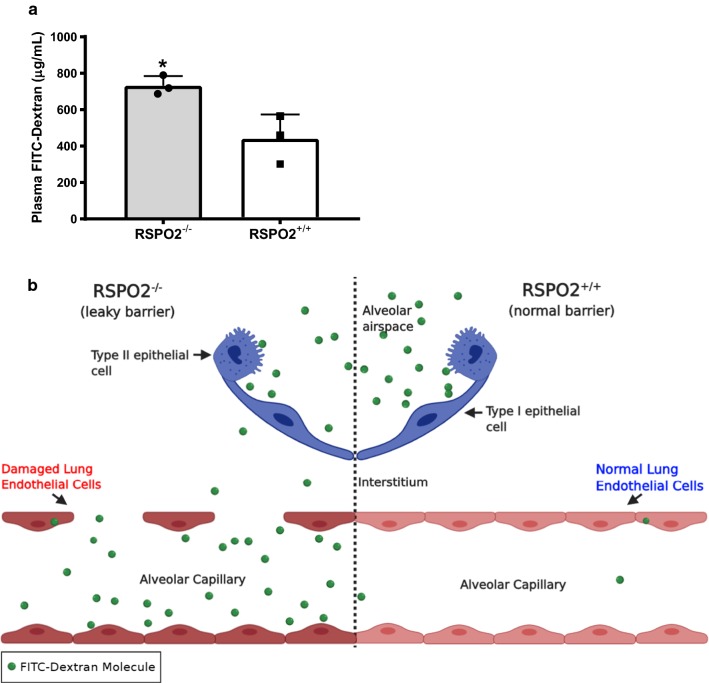
RSPO2 deletion increases lung permeability, as determined by a FITC-dextran assay. **a** A significant increase in average plasma dextran concentration (ug/ml) was observed in RSPO2^−/−^ mice compared to identically treated RSPO2^+/+^ mice 48 h after TM administration. **b** The movement of FITC-Dextran into normal alveolar capillaries (right side) and damaged alveolar capillaries (left side) during a murine model of endothelial cell barrier damage. Shown are the two crucial cell barriers, epithelial cells and endothelial cells. After intranasal instillation of FITC-Dextran solution, FITC-labeled molecules travel through the interalveolar space and interstitium into alveolar capillaries. Each dot represents the average of each experimental group in each of n = 3 independent experiments. Statistical significance was demonstrated when mice from the control group and experimental group were averaged within each of the independent experiments to control for the inherent variability in FITC-Dextran administration. When individual mice were pooled regardless of experiment, the results show a very similar trend as the averaged group, though not statistically significant. * = A P value of less than 0.05 was considered significant

Because neutrophils must exit circulation through the vasculature before translocation into the alveolar lumen [13], we hypothesized that RSPO2 deletion might induce endothelial disruption to facilitate the observed egress of neutrophils into the bronchoalveolar space. To assess lung permeability resulting from endothelial disruption, we administered FITC-dextran via intranasal instillation [16–18] and measured fluorescence in blood plasma after 30 min. A significant increase in plasma dextran concentration was observed in RSPO2^−/−^ mice compared to identically treated RSPO2^+/+^ mice (Fig. 3a, b). Taken together, these data indicate that RSPO2 deletion increases lung barrier permeability.

### Discussion

While RSPO2 expression in the embryonic lung mesenchyme is essential for normal lung development [2], whether RSPO2 expression in the adult lung is relevant in tissue homeostasis or repair has not been investigated. Our studies indicate an unexpected and biologically important role for RSPO2 in the lung as a regulator of neutrophil homeostasis and endothelial barrier function. Deletion of RSPO2 induces vascular leak and neutrophil accumulation in the airspace, indicating a novel role for R-Spondin signaling in these contexts.

To the best of our knowledge, RSPO2 has not been previously implicated in neutrophil homeostasis/chemotaxis. Given the well described role of R-Spondins in potentiating Wnt signaling, we presume dysregulation of Wnt is the likely driver behind this phenotype. For example, Wnt5a is known to activate noncanonical Wnt pathways and activate neutrophil chemotaxis [20], and although involvement of R-spondins has not been investigated, our work supports their possible involvement.

RSPO2’s role in regulation of vascular permeability is not entirely without precedent. In the adult gastrointestinal tract, another RSPO family member, RSPO3, tightens endothelial cell junctions, limiting fluid egress from the circulation [21]. Given high expression of RSPO2 in the developing lung, RSPO2 may also play a role in the dynamic regulation of microvascular permeability that occurs at birth during the transition to air breathing [22, 23]. It is worth considering whether RSPO2, by increasing barrier integrity, may act to counterbalance other factors which decrease integrity/induce permeability, akin to how endothelin and nitric oxide act as natural counterparts to regulate vasoconstriction and vasodilation, respectively [24].

While these studies highlight potentially important new roles for RSPO2, there are many outstanding questions which require further study. First, we presume that the combination of barrier dysfunction and a second mechanism, likely involving neutrophil chemokine dysregulation, explains the appearance of neutrophils in BALF as opposed to nonspecific accumulation of circulating immune cells. Further studies are also needed to understand whether neutrophils are being actively recruited or whether they arrive in the alveolar space passively. Moreover, because we employed a broadly expressed Cre driver, whether the phenotypes described here are cell autonomous or non-autonomous is unknown. Based on developmental studies [2, 25] we presume the lung mesenchyme is the predominant source of RSPO2 which acts primarily in a paracrine fashion (i.e. from mesenchymal cells to endothelial cells and hematopoietic cells), but this should be formally investigated. It is also possible that autocrine RSPO2 deletion in the endothelium itself leads to the vascular phenotype. Likewise, RSPO2 deletion in the neutrophils themselves could cause spurious activation. Our findings here indicate careful, cell type-specific studies should be performed to elucidate the range of RSPO2 functions in the adult lung and beyond.

Further investigations at the molecular level will be necessary to shed more light on the exact molecular mechanisms by which RSPO2 may regulate neutrophil migration, chemoattractant responsiveness, and basal lung barrier function. Ultimately, these initial findings should seed larger efforts to elucidate specific roles for RSPO2 in lung homeostasis and disease.

## Limitations

Since we utilized a global UBC-CreERT2 model to delete RSPO2, our studies cannot identify the most relevant cellular producers of RSPO2. Moreover, RSPO2 itself is a secreted, diffusible factor. As such, it is difficult to know either the cellular source of RSPO2 or the cell types(s) responding to RSPO2 signals. Future studies should use careful lineage-specific deletion to address these limitations.

## Data Availability

All data generated or analyzed during this study is included in this published article. Accompanying unprocessed, raw data is available from the corresponding author on reasonable request.

## Abbreviations

4-OHT: 4-Hydroxytamoxifen
ARDS: Acute respiratory distress syndrome
BALF: Bronchoalveolar lavage fluid
DMEM: Dulbecco’s modified Eagle’s medium
DMSO: Dimethyl sulfoxide
FITC: Fluorescein isothiocyanate
ICC: Immunocytochemical
LGRs: Leucine-rich repeat-containing G-protein coupled receptors
MPO: Myeloperoxidase
P/S: Penicillin-streptomycin
qPCR: Quantitative PCR
RSPO2: R-spondin 2
RSPO2^−/−^: R-spondin 2-deficient
RSPO2^+/+^: R-spondin 2 control
TM: Tamoxifen
UBC-CreERT2: Inducible Cre
